# Integrating Reinforcement Learning and Monte Carlo Tree Search for enhanced neoantigen vaccine design

**DOI:** 10.1093/bib/bbae247

**Published:** 2024-05-20

**Authors:** Yicheng Lin, Jiakang Ma, Haozhe Yuan, Ziqiang Chen, Xingyu Xu, Mengping Jiang, Jialiang Zhu, Weida Meng, Wenqing Qiu, Yun Liu

**Affiliations:** MOE Key Laboratory of Metabolism and Molecular Medicine, Department of Biochemistry and Molecular Biology, School of Basic Medical Sciences and Shanghai Xuhui Central Hospital, Fudan University, 131 DongAn Road, Shanghai, 200032, China; State Key Laboratory of Medical Neurobiology and MOE Frontiers Center for Brain Science, Institutes of Brain Science, Fudan University, 131 DongAn Road, Shanghai, 200032, China; MOE Key Laboratory of Metabolism and Molecular Medicine, Department of Biochemistry and Molecular Biology, School of Basic Medical Sciences and Shanghai Xuhui Central Hospital, Fudan University, 131 DongAn Road, Shanghai, 200032, China; State Key Laboratory of Medical Neurobiology and MOE Frontiers Center for Brain Science, Institutes of Brain Science, Fudan University, 131 DongAn Road, Shanghai, 200032, China; MOE Key Laboratory of Metabolism and Molecular Medicine, Department of Biochemistry and Molecular Biology, School of Basic Medical Sciences and Shanghai Xuhui Central Hospital, Fudan University, 131 DongAn Road, Shanghai, 200032, China; State Key Laboratory of Medical Neurobiology and MOE Frontiers Center for Brain Science, Institutes of Brain Science, Fudan University, 131 DongAn Road, Shanghai, 200032, China; MOE Key Laboratory of Metabolism and Molecular Medicine, Department of Biochemistry and Molecular Biology, School of Basic Medical Sciences and Shanghai Xuhui Central Hospital, Fudan University, 131 DongAn Road, Shanghai, 200032, China; State Key Laboratory of Medical Neurobiology and MOE Frontiers Center for Brain Science, Institutes of Brain Science, Fudan University, 131 DongAn Road, Shanghai, 200032, China; MOE Key Laboratory of Metabolism and Molecular Medicine, Department of Biochemistry and Molecular Biology, School of Basic Medical Sciences and Shanghai Xuhui Central Hospital, Fudan University, 131 DongAn Road, Shanghai, 200032, China; State Key Laboratory of Medical Neurobiology and MOE Frontiers Center for Brain Science, Institutes of Brain Science, Fudan University, 131 DongAn Road, Shanghai, 200032, China; MOE Key Laboratory of Metabolism and Molecular Medicine, Department of Biochemistry and Molecular Biology, School of Basic Medical Sciences and Shanghai Xuhui Central Hospital, Fudan University, 131 DongAn Road, Shanghai, 200032, China; State Key Laboratory of Medical Neurobiology and MOE Frontiers Center for Brain Science, Institutes of Brain Science, Fudan University, 131 DongAn Road, Shanghai, 200032, China; MOE Key Laboratory of Metabolism and Molecular Medicine, Department of Biochemistry and Molecular Biology, School of Basic Medical Sciences and Shanghai Xuhui Central Hospital, Fudan University, 131 DongAn Road, Shanghai, 200032, China; State Key Laboratory of Medical Neurobiology and MOE Frontiers Center for Brain Science, Institutes of Brain Science, Fudan University, 131 DongAn Road, Shanghai, 200032, China; MOE Key Laboratory of Metabolism and Molecular Medicine, Department of Biochemistry and Molecular Biology, School of Basic Medical Sciences and Shanghai Xuhui Central Hospital, Fudan University, 131 DongAn Road, Shanghai, 200032, China; State Key Laboratory of Medical Neurobiology and MOE Frontiers Center for Brain Science, Institutes of Brain Science, Fudan University, 131 DongAn Road, Shanghai, 200032, China; Shanghai Xuhui Central Hospital, 366 North Longchuan Road, Shanghai, 200231, China; MOE Key Laboratory of Metabolism and Molecular Medicine, Department of Biochemistry and Molecular Biology, School of Basic Medical Sciences and Shanghai Xuhui Central Hospital, Fudan University, 131 DongAn Road, Shanghai, 200032, China; State Key Laboratory of Medical Neurobiology and MOE Frontiers Center for Brain Science, Institutes of Brain Science, Fudan University, 131 DongAn Road, Shanghai, 200032, China

**Keywords:** human leukocyte antigen, major histocompatibility complex, Reinforcement Learning, Monte Carlo Tree Search, neoantigen vaccine design

## Abstract

Recent advances in cancer immunotherapy have highlighted the potential of neoantigen-based vaccines. However, the design of such vaccines is hindered by the possibility of weak binding affinity between the peptides and the patient’s specific human leukocyte antigen (HLA) alleles, which may not elicit a robust adaptive immune response. Triggering cross-immunity by utilizing peptide mutations that have enhanced binding affinity to target HLA molecules, while preserving their homology with the original one, can be a promising avenue for neoantigen vaccine design. In this study, we introduced UltraMutate, a novel algorithm that combines Reinforcement Learning and Monte Carlo Tree Search, which identifies peptide mutations that not only exhibit enhanced binding affinities to target HLA molecules but also retains a high degree of homology with the original neoantigen. UltraMutate outperformed existing state-of-the-art methods in identifying affinity-enhancing mutations in an independent test set consisting of 3660 peptide–HLA pairs. UltraMutate further showed its applicability in the design of peptide vaccines for Human Papillomavirus and Human Cytomegalovirus, demonstrating its potential as a promising tool in the advancement of personalized immunotherapy.

## Introduction

The major histocompatibility complex (MHC) molecules, commonly referred to as human leukocyte antigen (HLA), present processed peptides on the cell surface for T cell recognition [1]. MHC class I molecules, primarily encoded by *HLA-A*, *B* and *C* genes, are expressed on the surface of almost all nucleated cells. These molecules mainly bind intracellular peptides that are 8–10 amino acids in length and present them to CD8+ T cells [2–4]. For eliciting a robust adaptive immune response, antigenic peptides must be presented at the surface of cells as peptide–HLA (pHLA) complexes for T cell recognition [5].

Tumor cells often harbor somatic mutations or virally introduced genetic sequences that are not present in healthy cells. A fraction of these aberrant sequences produce non-self-peptides termed neoantigens [6]. These can be presented by MHC class I molecules, leading toward CD8+ T cells-mediated cytotoxic tumor destruction. As neoantigens are unique to tumor cells [7], they are promising candidates for therapeutic cancer vaccines [8]. Recent clinical trials have underscored the feasibility and safety of personalized neoantigen vaccines in various cancers [9–11]. Yet, the high polymorphism of MHC molecules and the variability in binding affinity of neoantigens to different MHC alleles complicate the process of peptide vaccine design [12].

In the past few years, numerous *in silico* screening pipelines have been developed to identify the optimal peptide for peptide vaccine design. Among various criteria evaluated, most existing approaches involve searching for peptides with high binding affinity to specific HLA alleles using *in silico* binding affinity prediction tools [13–19]. A similar strategy has also been applied in the context of tumor neoantigen design [9, 10, 20–23]. However, the process of individually searching through numerous peptides using these tools is inefficient. Moreover, these methods predominantly rank neoantigen candidates based on their binding affinity to specific HLA alleles of patients, potentially overlooking candidates with lesser binding affinity but higher prevalence. Only a minor fraction of top-ranked antigens have proven their immunogenicity in experimental setups [24–26]. Triggering cross-immunity by utilizing peptide mutations that have enhanced binding affinity for target HLA molecules, while preserving their homology with the original antigen, can be a promising avenue for neoantigen vaccine design.

For an 8-mer antigen peptide, with 20 potential amino acids for each position, it can, in theory, yield 20^8^ candidate mutations, posing a significant challenge in identifying potentially effective mutations. Hence, developing an efficient approach to identifying affinity-boosting peptide mutations is of paramount importance. Recent advancements in this field include the automatically optimized mutated peptides (AOMP) program [27], published in 2022, which was based on TransPHLA [27], a transformer-based model for predicting pHLA binding affinity. However, AOMP utilizes rank-based heuristic approach, which interprets the attention matrices from TransPHLA [27] as an indicator of the significance of pHLA interactions. This can be misleading [28, 29] and may thus result in its imperfect performance.

Reinforcement Learning (RL) trains agents to optimize long-term rewards through their interactions with intricate environments, and it has found applications in various fields, including robotics [30], game playing [31], finance [32], mathematics [33] and beyond. Monte Carlo Tree Search (MCTS) [34, 35] is a heuristic search algorithm used for decision-making. Their combination has propelled several achievements, including in game playing like AlphaGo [36], AlphaGo Zero [37] and Muzero [38] and in protein engineering [39], highlighting their effectiveness in navigating complex scenarios. In this study, leveraging the synergies of RL and MCTS, we developed an algorithm that identifies peptide mutations with enhanced binding affinity to target HLA molecules while retaining high homology. Unlike available approaches such as AOMP, which utilizes the attention matrices for rank-based heuristic methods, we postulate that the inherent pattern within the attention matrices holds vital information for mutation selection. By integrating supervised training based on AOMP’s mutation strategies with unsupervised RL and MCTS, we evaluated mutation results in an independent test set comprising 3660 pHLA pairs. By applying UltraMutate to two candidate peptides derived from human papillomavirus (HPV) and human cytomegalovirus (HCMV), we demonstrated the versatile applicability of our methodology in peptide vaccine design.

## Methods

We proposed a novel method called UltraMutate, which consists of supervised learning of the SL policy network, reinforcement learning of the RL policy network and MCTS to identify promising affinity-enhancing peptide mutations for a given non-binding pHLA pair.

### Supervised learning using the AOMP dataset

To identify affinity-enhancing peptide mutations for a given pHLA pair, we could theoretically utilize RL algorithms from scratch, without relying on prior expert knowledge. However, this would require extensive exploration within the entire mutation action space. Thus, our initial step involved using a supervised learning (SL) policy network to learn the mutation strategy of AOMP [27]. We employed the AOMP algorithm to mutate peptides that did not bind to their corresponding HLA molecules within TransPHLA’s pHLA dataset [27]. This yielded a dataset of approximately 1.8 million pHLA pairs with around 2.7 million AOMP mutations (the AOMP dataset). The AOMP dataset was subsequently divided into training, validation and test sets in an 8:1:1 ratio.

**Figure 1:**
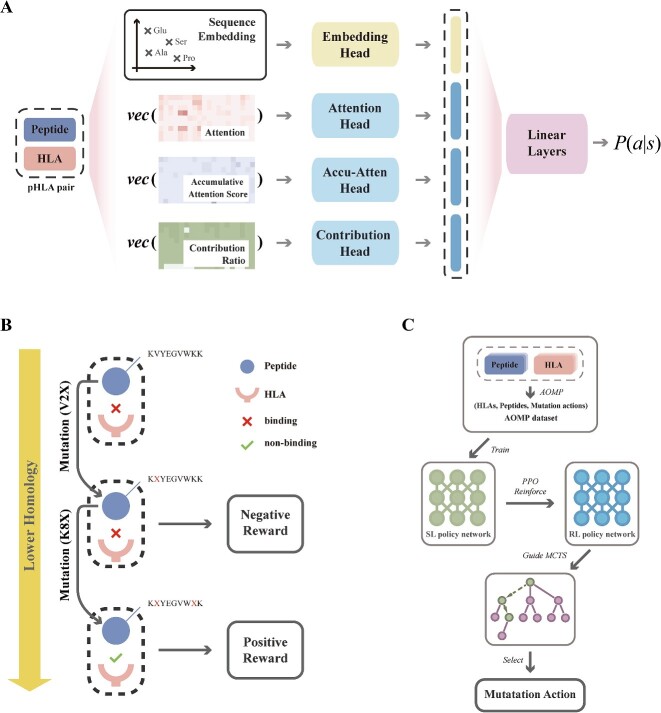
Overview of UltraMutate’s architecture and workflow. (**A**) For a given pHLA pair, the SL policy network takes embedded sequences and flattened vectors from three distinct attention matrices as input. It uses a linear layer to combine features from four input heads to generate a conditional probability distribution $P\left(s\right|a)$ for all possible mutation actions. (**B**) The interactive environment for the agent, where rewards are given based on the binding probability of the mutated peptide with the HLA molecule and the homology between the mutated and original peptides. (**C**) UltraMutate’s overall workflow includes: (1) supervised learning with the AOMP dataset; (2) fine-tuning the policy network using RL with PPO; and (3) integrating with an MCTS algorithm to determine the final mutation action.

Specifically, three distinct attention matrices for a given pHLA pair were extracted through TransPHLA’s forward pass (Fig. 1A): (1) the pHLA attention score matrix, obtained from the forward pass of TransPHLA with the specific pHLA pair as input; (2) the accumulative attention score matrix, representing the HLA molecule’s aggregate scores for all peptides matching the length of the target peptide; and (3) the contribution ratio matrix, representing the relative contribution of each amino acid site compared to all possible peptide amino acid sites. Instead of ranking segments in these attention matrices as was done in AOMP, we use neural networks to extract useful information from these attention matrices, along with the embedded matrix of sequences for peptide and HLA (Fig. 1A).

The SL policy network parameterized by $\sigma$ (denoted as ${p}_{\sigma }$) consists of four heads, each processing one of four types of observed contents, collectively referred to as state $s$. A softmax layer at the end provides a conditional probability distribution for all potential mutation actions, which is a 300-dimensional vector designed to represent the mutation probabilities for 20 amino acids across a 15-mer peptide.

We employed stochastic gradient ascent to maximize the likelihood of the mutation actions $a$ that AOMP selected in state $s$.


$$ \Delta \sigma \propto \frac{\partial \log{p}_{\sigma}\left(a| s\right)}{\partial \sigma } $$

### Reinforcement learning of the policy network

The second step in the process is aimed to improve the policy network by policy gradient RL. This process yields the RL policy network, which has the same architecture and initial parameters as the SL policy network. In each iteration, the RL policy network selects mutation actions for randomly sampled pHLA pairs. Each mutation action is assessed using a reward function. The design of reward function takes two factors into account: (1) the extent of change in binding probability brought by a mutation (obtained through TransPHLA [27]) and (2) the degree to which the mutation alters the peptide’s homology (Fig. 1). Based on these, the reward function ($r$) was formulated as:


$$ {\displaystyle \begin{array}{ll}& r=-{r}_f+\ln\ A+S\\{}& S=\left\{\begin{array}{@{}ll}\ 10&\ \mathrm{if}\ A\ge 0.5\\{}\ 0&\ \mathrm{if}\ A<0.5\end{array}\right.\end{array}} $$

Here, the term $-{r}_f$is a fixed negative value of −1 to encourage the model to identify the desired mutated peptide with minimal mutation actions. The variable $A$ is the binding probability for the current pHLA pair as predicted by TransPHLA. The variable $S$ is a reward that takes the constant value of 10 when the mutated peptide’s binding probability predicted by TransPHLA meets a predefined threshold of 0.5, in line with TransPHLA’s default demarcation between peptides that bind and those that don’t.

We implemented Proximal Policy Optimization (PPO) [40] as the RL algorithm to train the RL policy network (denoted as${p}_{\theta }$), tuned from the trained SL policy network. The RL policy network’s parameter, $\theta$, is updated using stochastic gradient descent to maximize the objective $L$.


$$ L\left(s,a,{\theta}_k,\theta \right)=\min \left(\frac{p_{\theta}\left(a| s\right)}{p_{\theta_k}\left(a| s\right)}{A}^{p_{\theta_k}}\left(s,a\right),g\left(\epsilon, {A}^{p_{\theta_k}}\left(s,a\right)\right)\right), $$

where


$$ g\left(\epsilon, A\right)=\left\{\begin{array}{@{}l}\ \left(1+\epsilon \right)A\ A\ge 0\\{}\ \left(1-\epsilon \right)A\ A\le 0\end{array}\right. $$

The advantage ${A}^{p_{\theta }}\left(a\right|s)$ is the difference between the Q-value ${Q}^{p_{\theta }}\left(s,a\right)$, the expected return of selection action $a$ in state $s$ and the value ${V}^{\varphi_k}(s)$, the predicted return of state $s$ by the critic network parameterized by $\varphi$ in the *k*-th iteration. $\epsilon$ is a hyperparameter that we set at 0.2, as used in the previous paper [40].

### MCTS

After fine-tuning the RL policy network through 15 000 mini-batches of training, we combine it with a modified MCTS [34, 35] algorithm to select a mutation by lookahead search. Starting from the root state, which represents the original non-binding peptide–HLA pair, the search tree is expanded through successive mutation actions selected from a given state. In this search tree, each node representing state *s* contains edges $\left(s,a\right)$ for all legal mutation actions $a$. Each edge stores a set of statistics,


$$ \left\{P\left(s,a\right),N\left(s,a\right),R\left(s,a\right),U\left(s,a\right),u\left(s,a\right)\right\} $$

where $P\left(s,a\right)$ is the prior probability given by the forward pass of the RL policy network and $N\left(s,a\right)$is the total number of visits to the edge $\left(s,a\right)$. The rollout value, $R\left(s,a\right)$, is defined by $R\left(s,a\right)=k\ln (H)+\beta$, where *H* is the homology score of the mutated peptide in the terminal state ${s}_T$ given a sequence of actions ${a}_{t\dots T}$ sampled from the RL policy network ${p}_{\theta }$at time steps from *t* to *T* (the terminal time step). We set constants *k* and *β* at 5 and 2, respectively. The discounted rollout value for an edge is denoted as $U\left(s,a\right)$, calculated by $U\left(s,a\right)=\frac{1}{N\left(s,a\right)}R\left(s,a\right)$. The term $u\left(s,a\right)$ is defined as the following using a variant of the PUCT algorithm [41]:


$$ u\left(s,a\right)={c}_{puct}P\left(s,a\right)\frac{\sqrt{\sum N\left(s,\cdot \right)}}{1+N\left(s,a\right)} $$

At each of these time steps *t*, an action ${a}_t$ is selected to maximize the sum of $U\left({s}_t,a\right)$ and $u\left({s}_t,a\right)$, formalized as:


$$ {a}_t=\underset{a}{\mathrm{argmax}}\left(U\left({s}_t,a\right)+u\left({s}_t,a\right)\right) $$

The remaining stages of the searching process, specifically expansion, evaluation and backpropagation, conform to the established MCTS method [34, 35]. Deploying this variant of MCTS on each pHLA pair, with a search depth of 10 and 60 simulation iterations, produces the findings detailed in the Results section.

The overall workflow of the mutation algorithm integrating the above policy networks and MCTS is illustrated in Fig. 1C.

### Structure prediction and molecular docking

AlphaFold2 [42] was utilized to predict the three-dimensional structures of the peptide and HLA molecule based on their sequence information. Following the prediction, molecular docking was performed using the ClusPro server [43–46], which enabled us to analyze the bonding interactions within the pHLA complex under consideration. The docking results generated by ClusPro were then visualized using PyMOL [47], allowing for a detailed analysis of the interactions between the amino acids of the peptide and HLA molecule.

### Peptide competitive binding assay for HLA-A molecules

The competitive peptide binding assay was performed as previously described [48] with minor modifications. Briefly, HMy2.CIR cell lines expressing HLA-A^*^11:01 [49] were collected and then washed with ice-cold elution buffer (0.131-M citric acid, 0.061-M Na_2_HPO_4_, pH 3.3 and filtered through a 0.22-μm filter) for 1 min, which was followed by immediate neutralization with ice-cold IMDM-2 medium supplemented with 0.5% bovine serum albumin (BSA). After centrifugation, the cells were resuspended to a density of 1 × 10^6^ cells/ml in IMDM-2 medium with 1 μg/ml β_2_M. Subsequently, 25 μl of either an unlabeled competitor original or a mutated peptide (5 or 15 μM) were added to the wells of a 96-well U-bottom plate, together with 25 μl of an fluorescein isothiocyanate (FITC)-labeled reference peptide (KVFPKALINK) at 300 nM. The plate was then incubated for 24 h at 4°C in darkness. Control wells lacking competitor test peptides (the negative control) and those with an unlabeled positive control peptide (QVPLRPMTYK) were both included.

After incubation, cells were centrifuged at 600 rpm for 5 min at room temperature and then washed twice with 100 μl of ice-cold PBS containing 0.5% BSA. The cell pellets were then resuspended in 150 μl of PBS for flow cytometry analysis. The competitive inhibition percentage at 5 μM (5-μM inhibition %) or 15 μM (15-μM inhibition %) was calculated using the following formula: [1 − (sample % − background %)/(max % − background %)] × 100% [49]. The term sample % refers to the percentage of FITC-positive cells in the experimental well; the term background % is the percentage of FITC-positive cells in the no-peptide control well and the term max % is the percentage of FITC-positive cells in wells without competitor test peptides. The IC50 value indicates the concentration of unlabeled competitor peptide needed to inhibit 50% of the FITC-labeled reference peptide’s binding, with an IC50 < 5 μM (5-μM inhibition >50%) indicating a high binding affinity, an IC50 between 5 and 15 μM (5-μM inhibition <50% and 15-μM inhibition >50%) indicating an intermediate binding affinity and an IC50 > 15 μM indicating low or no binding affinity (5-μM inhibition of 20–50% or 15-μM inhibition of 30–50% means a low binding affinity; 5-μM inhibition of <20% or 15-μM inhibition of <30% means no binding affinity).

## Results

### Evaluation of UltraMutate with other state-of-the-art methods

To assess UltraMutate’s efficacy in identifying peptide mutations with enhanced binding affinity while minimizing the loss of homology with the original one, we conducted a comparative analysis with the AOMP algorithm and the RL-based approach, PepPPO [50]. We evaluated an independent dataset of 3660 negative pHLA pairs, covering all 366 different HLA–peptide length combinations (peptide lengths varying from 8 to 14) available in TransPHLA’s independent test set [27]. We employed this peptide selection process to ensure a thorough examination of both methods across a spectrum of various pHLA combinations, which was originally introduced in the evaluation of AOMP [27]. UltraMutate, AOMP and PepPPO were utilized in parallel to identify potential mutations capable of increasing the peptide’s binding affinity to the target HLA molecules. The binding affinity of the original and mutated peptides was assessed using TransPHLA. Mutations were deemed successful if they resulted in a binding probability surpassing TransPHLA’s default threshold of 0.5 in less than four mutation actions. Among all tested pHLA pairs, UltraMutate achieved a success rate of 99.45%, surpassing AOMP’s 99.10% and PepPPO’s 72.98% (Table 1).

**Table 1:** Comparison of UltraMutate, AOMP and PepPPO using 3660 test samples

	UltraMutate	AOMP	PepPPO
Successful samples	3640 (99.45%)	3627 (99.10%)	2671 (72.98%)
Samples w. 1 mutation site	1855 (50.68%)	1664 (45.46%)	174 (4.75%)
Samples w. 2 mutation sites	1572 (42.95%)	1563 (42.70%)	771 (21.07%)
Samples w. 3 mutation sites	198 (5.41%)	336 (9.18%)	964 (26.32%)
Samples w. 4 mutation sites	15 (0.41%)	64 (1.75%)	762 (20.82%)
Failed samples	20 (0.55%)	33 (0.90%)	989 (27.02%)

In 93.6% of pHLA pairs, UltraMutate identified peptide mutants with no more than two mutation sites, maintaining a homology score that exceeded 0.8. This performance surpasses AOMP’s performance of 88.2%. In contrast, PepPPO identified only 25.8% of the pHLAs with two or fewer mutation sites. This discrepancy can be attributed to PepPPO’s training method, which focuses on optimizing peptide–HLA binding affinity while not considering the preservation of homology. Therefore, UltraMutate provides a more effective mutation strategy for conserving the homology of the original peptide. UltraMutate does take longer computational time than AOMP and PepPPO in optimizing peptides (Supplementary Table 1), primarily due to its implementation of MCTS, which entails running searches using trained neural networks. In real-world applications, it remains efficient and practical, especially when deployed in parallel.

### UltraMutate’s strategic advantage over attention weights–based heuristics

We delved deeper into the factors contributing to UltraMutate’s better performance compared to the rank-based heuristic approach of AOMP. Unlike UltraMutate, AOMP relies on attention weights to focus on specific input segments during mutation processes. However, interpreting these attention weights as direct contributions can lead to inaccuracies or potential misinterpretations [28, 29]. Hence, a heuristic algorithm derived from such interpretations may not always yield the best results. To evaluate UltraMutate’s effectiveness over such heuristic strategies, we examined three pHLA pairs as case studies.

**Figure 2:**
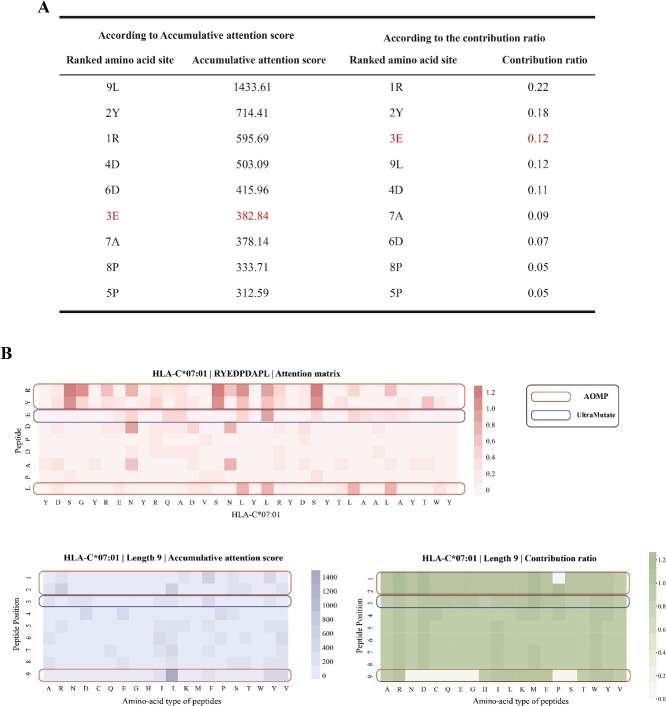
Comparative analysis of UltraMutate and AOMP for the pairing of HLA-C^*^07:01 with the peptide RYEDPDAPL. (**A**) The summary table showing a ranking of amino acid positions determined by AOMP based on the accumulative attention score and contribution ratio for the original peptide, where UltraMutate identified another mutation site (3E) that does not have the highest accumulative attention score or contribution ratio. (**B**) Graphical representations of amino acid positions in three attention matrices for the pHLA pair extracted through TransPHLA’s forward pass. Mutation sites selected by AOMP were marked by the corresponding circles.

For the pairing of the HLA allele HLA-C^*^07:01 with the peptide RYEDPDAPL, derived from Human gammaherpesvirus 4, the heuristic AOMP method prioritized the mutation sites of leucine at position 9 (9L), tyrosine at position 2 (2Y) and arginine at position 1 (1R) as the first mutation sites, guided by the high accumulative attention scores and contribution ratios corresponding to these peptide positions with the HLA molecule (Fig. 2). AOMP’s selection on mutation sites was influenced by two main factors: the accumulative attention score from all peptides with a length of nine amino acids and the contribution ratio from the specific attention matrix for the pHLA pair under study. After the first step of mutation, AOMP identified some more mutation sites in order to reach the required binding affinity level, resulting in the final peptide that met the requirements within two mutation sites in total.

UltraMutate, however, identified the optimal mutation action, E3Y, on an alternative mutation site (3E) that did not rank highest in either accumulative attention score or contribution ratio (Fig. 2). This strategic selection by UltraMutate highlights its capability to surpass heuristic methods like AOMP in pinpointing optimal mutation sites, thus optimizing the peptide’s binding affinity without the need for extra mutations. This instance is not unique but is one of many where UltraMutate consistently outperformed the attention weights–based strategy of AOMP (Supplementary Figures 1 and 2). These examples provide evidence of UltraMutate’s superior ability in identifying optimal mutation actions by leveraging the inherent pattern within the attention matrices.

### Application of UltraMutate in HPV and HCMV peptide vaccines

UltraMutate’s efficacy was further evaluated in the context of potential peptide vaccine design. Human papillomavirus (HPV) is the most prevalent viral infection of the reproductive tract, with 99.7% of cervical cancers attributable to the virus [51]. Extensive clinical trials have provided compelling evidence that HPV vaccination can significantly reduce the incidence of cervical cancer [52]. In particular, HPV type 52 has a notable incidence in East Asia, accounting for 6.5% of invasive cervical cancers in China and 8.7% in Japan [53]. The high prevalence of this HPV type underscores an urgent need for its effective therapeutic vaccines.

A peptide sequence, ELQRREVYK, originating from the E6 oncoprotein of HPV type 52, has been recognized as an HLA-A^*^11:01-restricted peptide through an *in vitro* peptide-binding assay [54]. However, the binding affinity predicted by NetMHCpan [55] for this pHLA pair is classified as weak, suggesting its suboptimal efficacy. Consistently, this pHLA pair does not elicit positive T-cell responses, as evidenced by its underperformance in the interferon-gamma enzyme-linked immunospot (IFN-γ ELISPOT) assay [54]. Considering the high prevalence of HLA-A^*^11:01 allele (21.143%) in the Chinese population [56], identifying mutations of the ELQRREVYK peptide with enhanced binding affinity to HLA-A^*^11:01 that elicit a robust cross-immune response could have significant clinical implications.

**Figure 3:**
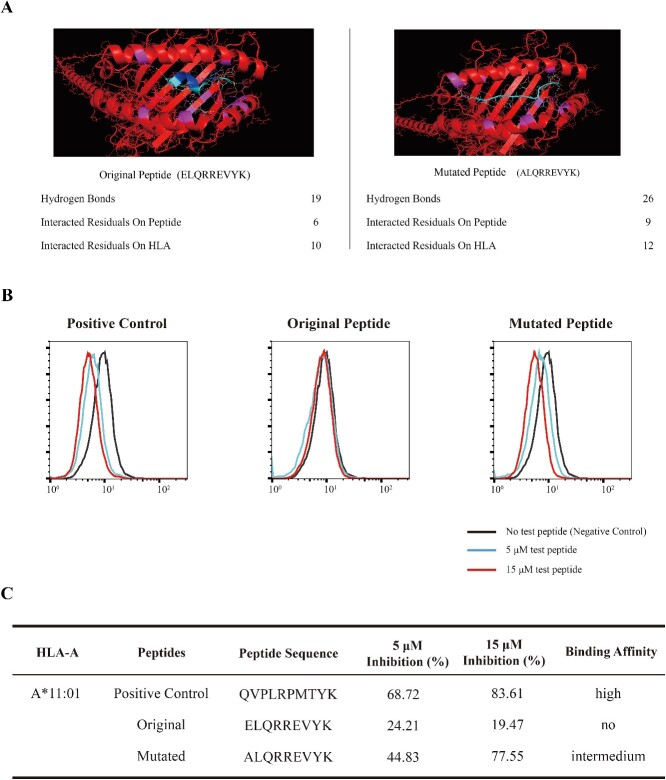
Binding evaluation of the pHLA complexes for HPV peptides. (A) Visualization of the pHLA complexes for the original (ELQRREVYK) and mutated (ALQRREVYK) peptides using PyMOL, with the statistics of the bonding interactions at the bottom. (**B**) Flow cytometry results show a leftward shift in the fluorescence intensity for the mutated peptide (ALQRREVYK), indicating its enhanced binding affinity to the HLA-A^*^11:01 allele in contrast to the original peptide (ELQRREVYK), which does not exhibit a discernible shift. The negative control is the experiment without competitor peptides, and the positive control is the one with an unlabeled positive control peptide. (**C**) Summarized binding affinity and competitive inhibition percentages of peptides with the HLA-A^*^11:01 allele.

We employed UltraMutate to this task, which successfully identified a single mutation, substituting the first amino acid from E to A (E1A). This mutation yielded the peptide ALQRREVYK, which was subsequently predicted as a strong binder to HLA-A^*^11:01 by both NetMHCpan and TransPHLA (Table 2). The mutated peptide retained substantial similarity to the original peptide, with a homology score of 0.89. Molecular docking studies of the original (ELQRREVYK) and mutated (ALQRREVYK) peptides with HLA-A^*^11:01 were conducted using the ClusPro online server [43–46]. The statistics of contact residues and the structure of the binding complex visualized with PyMOL [47] further revealed enhanced binding affinity of the mutated peptide (Fig. 3A). In contrast, AOMP was unable to generate a mutation within a single site and required at least two mutation sites to produce a mutated peptide with a diminished homology score of 0.78.

**Table 2:** Evaluation of binding potential for peptides derived from HPV and HCMV

	Peptide sequence	HLA allele	NetMHCpan evaluation		TransPHLA evaluation	
			%Rank	Bind level	Binding probability	Binding result
Original peptide (HPV)	ELQRREVYK	HLA-A^*^11:01	1.864	Weak binder	0.003	Non-binding
Mutated peptide (HPV)	ALQRREVYK	HLA-A^*^11:01	0.408	Strong binder	0.995	Binding
Original peptide (HCMV)	KEVNSQLSL	HLA-B^*^15:10	1.002	Weak binder	0.000	Non-binding
Mutated peptide (HCMV)	FEVNSQLSL	HLA-B^*^15:10	0.269	Strong binder	0.993	Binding

%Rank: rank of the predicted binding score compared to a set of random natural peptides introduced by NetMHCpan. Peptides will be identified as weak binder if the %Rank is above the threshold of the strong binders (by default, 0.5%) but below the threshold of the weak binders (by default, 2%). Binding probability: the binding probability predicted by TransPHLA. Peptides will be identified as non-binding to the HLA allele if the binding probability is below the default threshold 0.5.

To further confirm the binding affinity enhancement, we performed a competitive binding assay [48, 49] by introducing the original or mutated peptides to HMy2.CIR cells expressing HLA-A^*^11:01 [49]. Flow cytometry analysis revealed a leftward shift in the fluorescence peak for the mutated peptide (Fig. 3B), indicating its efficient binding to the HLA-A^*^11:01 allele. In contrast, the original peptide showed no discernible shift (Fig. 3B). The binding affinities and IC50 values are summarized in Fig. 3C, with the mutated peptide displaying an intermediate binding affinity within the 5–15-μM range.

We applied UltraMutate to another candidate peptide, KEVNSQLSL from the IE1 protein of HCMV [57, 58], targeting the HLA-B^*^15:10 allele. The proposed single-site mutation of the first amino acid K to F (K1F) led to a substantial increase in binding as predicted by both NetMHCpan and TransPHLA (Table 2). The mutated peptide, FEVNSQLSL, exhibited an impressive homology score of 0.89. However, AOMP suggested a peptide with two mutation sites, resulting in a lower homology score of 0.78. The structural representations and statistics of contact residues of the binding complexes for this pHLA pair are also depicted in Supplementary Figure 3. Collectively, these findings underscore UltraMutate’s efficacy in scenarios of peptide vaccine design.

## Discussion

In recent years, several *in silico* screening pipelines have emerged to predict the binding affinity between the pHLA pair, which greatly facilitates the design of peptide vaccines. Notable tools, including NetMHCPan and NetMHCIIPan, [55] have been employed in the peptide selection processes for developing vaccines against pathogens such as *Staphylococcus saprophyticus* [19], MERS-CoV [14] and SARS-CoV-2 [13, 16, 17]. Additionally, HLAPred was utilized in the design of a peptide vaccine against *Dermatophagoides pteronyssinus* [18]. While these existing methods employ *in silico* binding affinity prediction tools to screen through numerous candidate antigens, the process of individually screening for peptides with a high binding affinity for specific HLA alleles remains inefficient. Moreover, the ranking of candidate peptides is predominantly based on their binding affinity, which may neglect candidate pHLA pairs with a higher prevalence of HLA molecules. Exploring the potential of triggering cross-immunity by screening peptide mutations with enhanced binding affinity to target HLA molecules, while preserving homology with the original one, offers another option, which can be easily achieved with UltraMutate. Through the integration of UltraMutate into the existing pipeline of vaccine development, we envision that it can not only enhance the efficiency of vaccine candidate selection but also streamline the path toward clinical translation by prioritizing peptides with greater therapeutic potential.

By comparing with the rank-based heuristic approach of AOMP, we revealed potential reasons leading to the better performance of UltraMutate. AOMP relies on attention weights to identify input segments for mutation actions. In the optimization of a specific pHLA pair, AOMP analyzed various attention matrices to prioritize mutation actions based on their attention weights. This approach was based on the belief that the values of attention weights represent the rankings of mutation actions. However, simply interpreting these attention weights as direct contributions can be misleading [28, 29]. Hence, a heuristic algorithm derived from such interpretations may not always achieve optimal results.

Another recently published work PepPPO shares similarities with UltraMutate in utilizing RL to identify mutation actions that enhance peptide binding affinity for a given pHLA pair. However, we’ve demonstrated that UltraMutate significantly outperformed PepPPO in the independent test set. The difference in performance can be attributed to several factors. First, UltraMutate leverages valuable information from TransPHLA’s attention matrices. In contrast, PepPPO is trained from scratch without prior knowledge. Second, the reward function in PepPPO lacks the consideration for maintaining the homology of mutated peptides, which greatly impacts its ability in identifying peptide mutations with high homology scores. Finally, unlike UltraMutate, PepPPO does not utilize MCTS to enhance its mutation action policy, which may limit its effectiveness in exploring and exploiting the vast mutation landscape.

One important aspect to consider is the ability of UltraMutate to generalize across a wide range of HLA alleles. The training dataset of UltraMutate, derived from TransPHLA, is meticulously curated from prominent databases including IEDB [59], EPIMHC [60], MHCBN [61] and SYFPEITHI [62]. This dataset comprehensively encompasses 112 common HLA class I (40 HLA-A, 56 HLA-B and 16 HLA-C) allele types, effectively covering a substantial portion of the HLA diversity in population. In practical application, an individual typically possesses a total of six alleles across the *HLA-A*, *HLA-B* and *HLA-C* genes. Given the prevalence of these 112 *HLA* alleles within the population, it is highly probable that an individual will possess at least one of these alleles included in the dataset. Thus, we believe that UltraMutate can be generally used to identify mutated peptides capable of binding to a patient’s specific HLA alleles.

The strategy of integrating RL with MCTS within the UltraMutate algorithmic framework shows significant potential, likely beyond the demonstrated scenario of peptide–HLA binding enhancement. The algorithm’s adeptness at deciphering complex patterns makes it a versatile tool for tasks requiring a careful balance between biological function and molecular diversity. The efficacy of this algorithmic framework has been demonstrated in other works, notably a recent one that utilized integrated RL and MCTS for protein engineering [39]. By modulating the protein structure properties obtained from AlphaFold2, Wang *et al.* [39] illustrated the application of such strategy to the generation of proteins with desired properties. We envision that the strategic algorithmic foundation of UltraMutate will lead to advancements in a variety of fields, including drug discovery, genetic research and beyond.

UltraMutate’s capabilities, while advanced, also come with certain limitations that offer avenues for future improvement. The first limitation is the absence of structural information in UltraMutate’s input. It relies on sequences of peptides and HLA molecules without considering the three-dimensional configurations that are critical in determining the pHLA interactions. While prevailing binding prediction models predominantly focus on sequence information due to the inherent brevity of peptides binding to MHC, there exists potential benefit in incorporating structural data into the model. Several algorithms [63–65] have successfully incorporated structural information for protein design purposes. To address this, incorporating molecular dynamics simulations or structural modeling into UltraMutate could provide a more nuanced understanding of how mutations may affect the physical interactions between peptides and HLA molecules. Given that UltraMutate employs TransPHLA to evaluate the binding affinity of pHLA pairs, and TransPHLA performs well without structural data, the lack of this information may have a minimal impact on UltraMutate’s capabilities. Nonetheless, such integrative approaches may further refine the predictions, making them more robust and applicable to real-world scenarios.

Another limitation pertains to UltraMutate’s assessment of peptide homology solely based on sequence similarity, which may not always translate to biological equivalence. Furthermore, UltraMutate focuses on HLA binding affinity without direct consideration of whether the resultant pHLA pair will activate T cells and elicit a cross-immune response. To bridge this gap, future iterations of UltraMutate could integrate sequence analysis with structural epitope mapping, potentially employing algorithms that consider the interactions between peptide–HLA pairs and T cell receptors to predict the immunogenicity of peptides. Integration of T cell receptor modeling and epitope prediction tools into the UltraMutate framework could provide a more comprehensive assessment of the peptide’s ability to initiate an adaptive immune response, moving closer to a biologically relevant measure of peptide vaccines.

## Conclusion

In this work, we present UltraMutate, an algorithm that leverages the power of RL and MCTS to identify peptide mutations. UltraMutate not only enhances peptide’s binding affinity to target HLA molecules but also maintains a high degree of homology with the original peptide. Our results demonstrate that UltraMutate outperforms existing heuristic approaches like AOMP and RL-based approaches such as PepPPO in identifying mutations that may enhance the immunogenic potential of neoantigens. Using HPV and HCMV peptides as examples, UltraMutate demonstrated its efficacy in the development of peptide vaccines.

The integration of RL with MCTS equips UltraMutate with a robust mechanism for navigating the vast mutation landscape more effectively than other state-of-art competitors. The current version can be seamlessly integrated with the existing pipelines for peptide vaccine development and is a stepping stone toward a comprehensive peptide vaccine design tool that is designed to potentially elicit an immune response. This could represent a significant advancement in the field of neoantigen-based vaccine design and a promising step toward personalized cancer immunotherapy.

**Key Points**

- In the pursuit of effective neoantigen-based vaccines, we introduced UltraMutate, an innovative algorithm that leverages Reinforcement Learning (RL) and Monte Carlo Tree Search (MCTS), aiming to identify peptide mutations that not only demonstrate improved binding affinities to target HLA molecules but also maintain a significant degree of homology with the original peptide.
- UltraMutate utilized the attention information generated by TransPHLA to identify affinity-enhancing peptide mutations. Instead of the existing rank-based heuristic method like AOMP, UltraMutate uses a neural network to output the probability distribution of mutation actions and combines the policy networks with a modified MCTS to ensure its performance.
- Demonstrating superior performance on a held-out test set, UltraMutate validates its efficacy compared to existing methods such as AOMP and another RL approach, PepPPO, which lacks consideration for maintaining homology and does not involve a searching mechanism.
- UltraMutate showed its applicability in the design of peptide vaccines for Human Papillomavirus and Human Cytomegalovirus, demonstrating its potential as a promising tool in the advancement of personalized immunotherapy.

## Supplementary Material

Supplementary_Figures_bbae247

Supplementary_Table_1_bbae247

## Data Availability

The TransPHLA’s pHLA dataset was downloaded at: https://github.com/a96123155/TransPHLA-AOMP/tree/master/Dataset. All the codes used in this work are available at: https://github.com/YichengLin-lab/ultramutate.

## Author Biographies

**Yicheng Lin** is a PhD student at School of Basic Medical Sciences, Fudan University, China. He works on both the theory of deep learning, reinforcement learning and their applications in various biological questions.

**Jiakang Ma** is a PhD student at School of Basic Medical Sciences, Fudan University, China. His work focuses on the mechanisms of the MHC pathway during immunotherapy of cancers.

**Haozhe Yuan** is a master student at School of Basic Medical Sciences, Fudan University, China. He focuses on the applications of deep learning and machine learning on large-scale genomic data.

**Ziqiang Chen** is a PhD student at School of Basic Medical Sciences, Fudan University, China. He works on the applications of deep learning on medical images.

**Xingyu Xu** is a PhD student at School of Basic Medical Sciences, Fudan University, China. His primary focus is the bioinformatic analyses of large-scale genomic data.

**Mengping Jiang** is a read Resolution master student at School of Basic Medical Sciences, Fudan University, China. Her work focuses on the epigenetic regulation of MHC molecules.

**Jialiang Zhu** is a master student at School of Basic Medical Sciences, Fudan University, China. His work focuses on the epigenetic regulation of MHC molecules.

**Weida Meng** is a postdoctoral fellow at School of Basic Medical Sciences, Fudan University, China. He specializes in the fields of molecular mechanism of epigenetic regulation.

**Wenqing Qiu** is an associate professor at Shanghai Xuhui Central Hospital, Fudan University, China. Her major research interests include genetic and epigenetic regulation of MHC pathway during tumor immune escape.

**Yun Liu** is a professor at School of Basic Medical Sciences, Fudan University, China. More information can be found at the website of his group: http://Liulab.fudan.edu.cn.
