# Supplementary material for: Integrating Reinforcement Learning and Monte Carlo Tree Search for enhanced neoantigen vaccine design
Source: Brief Bioinform. 2024 May 20;25(3):bbae247. doi: 10.1093/bib/bbae247 (PMC11107383; doi:10.1093/bib/bbae247)
Supplement: Supplementary_Figures_bbae247 [file supplementary_figures_bbae247.pdf]

Supplementary Figure Legends:

Supplementary Figure 1: Comparative analysis of UltraMutate and AOMP for the pairing of HLA-A*26:01 with the peptide TLHGPTPLL.

Supplementary Figure 2: Comparative analysis of UltraMutate and AOMP for the pairing of HLA-C*06:02 with the peptide QKWTVEESEWV.

Supplementary Figure 3: Binding evaluation of the pHLA complexes for HCMV peptides. Visualization of the pHLA complexes for the original (KEVNSQLSL) and mutated (FEVNSQLSL) peptides using PyMOL, with the statistics of the bonding interactions at the bottom.

Supplementary Figure 1

a

According to Accumulative attention score		According to the contribution ratio	
**Ranked amino acid site**	**Accumulative attention score**	**Ranked amino acid site**	**Contribution ratio**
1T	834.18	1T	0.17
9L	767.13	9L	0.14
2L	595.99	2L	0.14
3H	364.78	8L	0.12
6T	307.93	4G	0.11
4G	245.70	3H	0.11
7P	199.16	7P	0.08
8L	168.49	6T	0.07
5P	141.88	5P	0.07

b

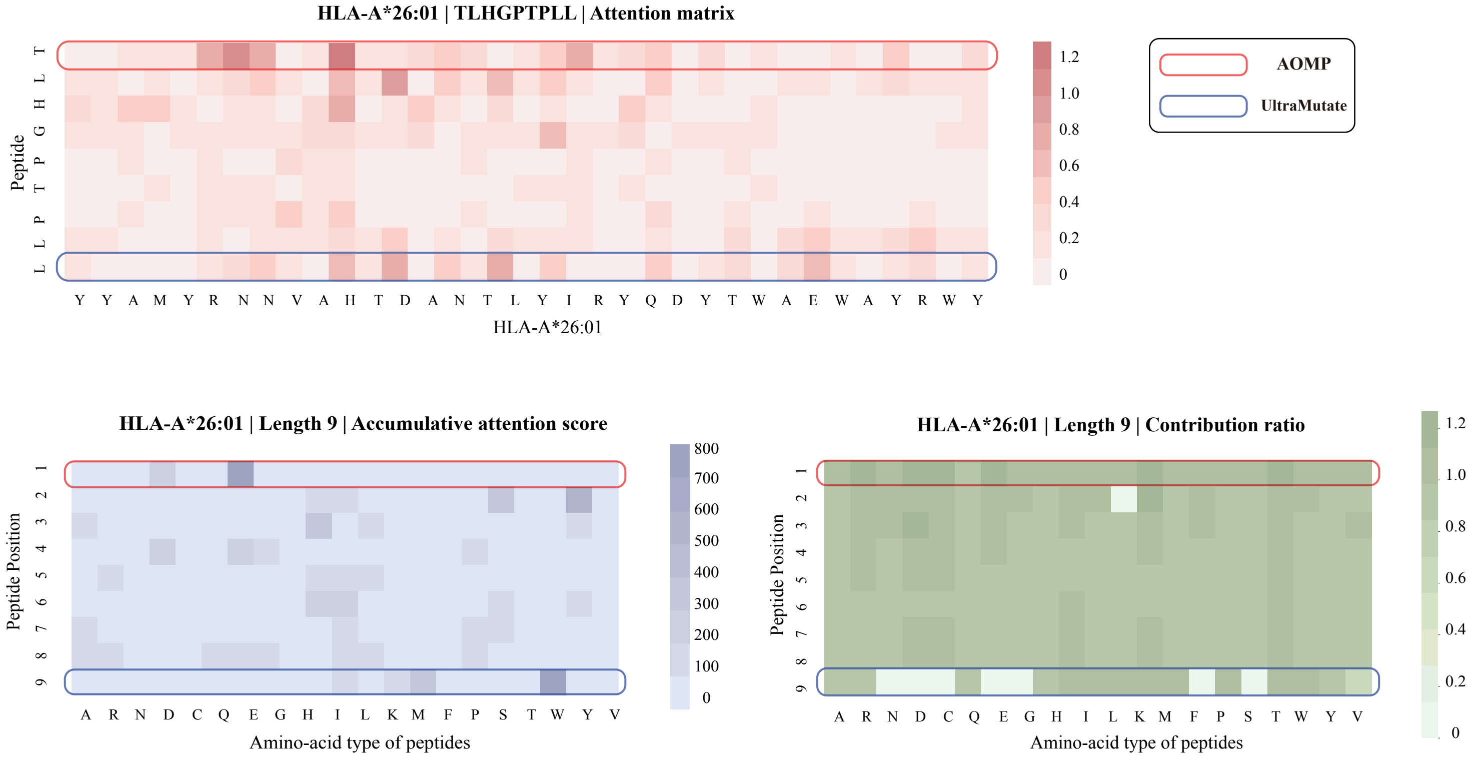

Supplementary Figure 2

a

According to Accumulative attention score		According to the contribution ratio	
**Ranked amino acid site**	**Accumulative attention score**	**Ranked amino acid site**	**Contribution ratio**
2K	48.94	3W	0.14
11V	39.72	2K	0.13
3W	20.47	1Q	0.11
4T	18.83	10W	0.11
9E	15.66	8S	0.09
1Q	15.53	9E	0.08
5V	12.82	11V	0.08
10W	11.47	6E	0.08
8S	11.41	7E	0.08
6E	11.32	5V	0.06
7E	10.60	4T	0.04

b

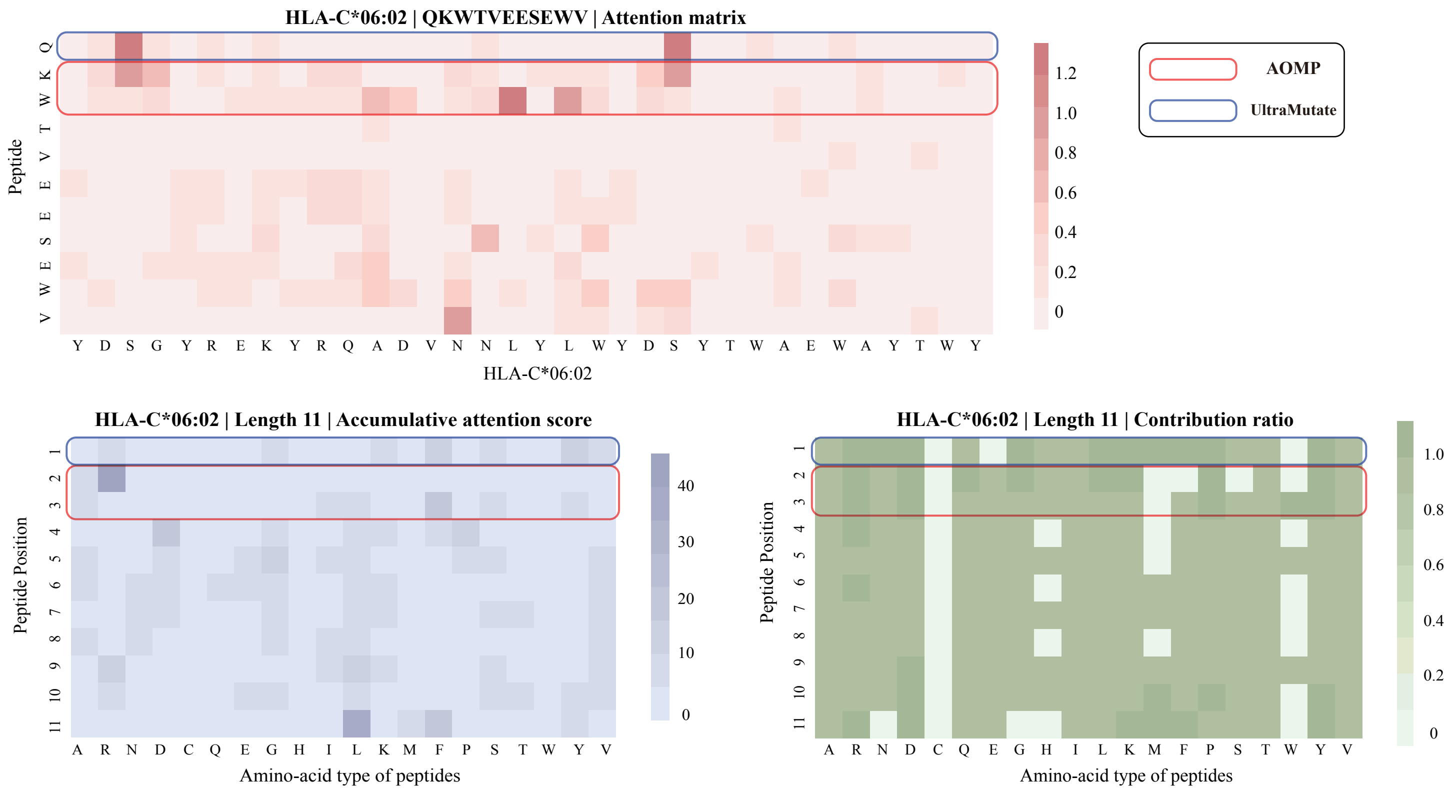

Supplementary Figure 3

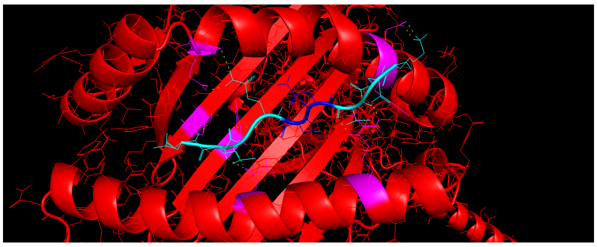

Original Peptide (KEVNSQLSL)

Hydrogen Bonds	15
Interacted Residuals On Peptide	7
Interacted Residuals On HLA	7

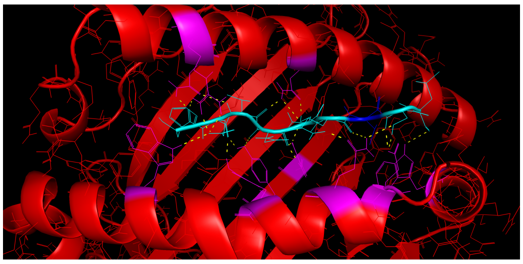

Mutated Peptide (FEVNSQLSL)

Hydrogen Bonds	21
Interacted Residuals On Peptide	8
Interacted Residuals On HLA	10
