# Supplementary material for: Integrating Reinforcement Learning and Monte Carlo Tree Search for enhanced neoantigen vaccine design
Source: Brief Bioinform. 2024 May 20;25(3):bbae247. doi: 10.1093/bib/bbae247 (PMC11107383; doi:10.1093/bib/bbae247)
Supplement: Supplementary_Table_1_bbae247 [file supplementary_table_1_bbae247.docx]

**Supplementary Table 1 | Computational time and memory comparison**

|  | **AOMP** | **PepPPO** | **UltraMutate** |
| --- | --- | --- | --- |
| **Time** | 0.59h | 1.07h | 2.01h |
| **Memory** | 1794.69MB | 3035.83MB | 1349.30MB |

**Time:** The total time spent when optimizing the 3660 pHLAs in the independent test set performed on a CPU with 256 cores and parallelization enabled.
**Memory:** Memory allocation during the implementation of the programs.
